# Human nitrobindin: the first example of an all‐β‐barrel ferric heme‐protein that catalyzes peroxynitrite detoxification

**DOI:** 10.1002/2211-5463.12534

**Published:** 2018-11-09

**Authors:** Giovanna De Simone, Alessandra di Masi, Fabio Polticelli, Paolo Ascenzi

**Affiliations:** ^1^ Department of Sciences Roma Tre University Italy; ^2^ National Institute of Nuclear Physics Roma Tre Section Italy; ^3^ Interdepartmental Laboratory for Electron Microscopy Roma Tre University Italy

**Keywords:** human nitrobindin, kinetics, peroxynitrite scavenging, protection of l‐tyrosine nitration

## Abstract

Nitrobindins (Nbs), constituting a heme‐protein family spanning from bacteria to *Homo sapiens*, display an all‐β‐barrel structural organization. Human Nb has been described as a domain of the nuclear protein named THAP4, whose physiological function is still unknown. We report the first evidence of the heme‐Fe(III)‐based detoxification of peroxynitrite by the all‐β‐barrel *C*‐terminal Nb‐like domain of THAP4. Ferric human Nb (Nb(III)) catalyzes the conversion of peroxynitrite to ${\text{NO}}_{3}^{-}$ and impairs the nitration of free l‐tyrosine. The rate of human Nb(III)‐mediated scavenging of peroxynitrite is similar to those of all‐α‐helical horse heart and sperm whale myoglobin and human hemoglobin, generally taken as the prototypes of all‐α‐helical heme‐proteins. The heme‐Fe(III) reactivity of all‐β‐barrel human Nb(III) and all‐α‐helical prototypical heme‐proteins possibly reflects the out‐to‐in‐plane transition of the heme‐Fe(III)‐atom preceding peroxynitrite binding. Human Nb(III) not only catalyzes the detoxification of peroxynitrite but also binds NO, possibly representing a target of reactive nitrogen species.

**Abbreviations**
*Cj*‐trHbP: *Campylobacter jejuni* truncated hemoglobin P
CL‐cyt*c*: cardiolipin‐bound cytochrome *c*
CM‐cyt*c*: carboxymethylated cytochrome *c*
Hb: hemoglobin
human Nb(III): ferric *Cj*‐trHbP
human SA‐heme: human serum heme‐albumin
*Ma*‐Pgb: *Methanosarcina acetivorans* protoglobin
Mb: myoglobin
*Mt*‐trHbN: *Mycobacterium tuberculosis* truncated hemoglobin N
Nb(III): ferric Nb
Nb: nitrobindin
NONOate: 1,1‐diethyl‐2‐hydroxy‐2‐nitroso‐hydrazine
NP: nitrophorin
*Ph*‐trHbO: *Pseudoalteromonas haloplanktis* truncated hemoglobin O

In living organisms, all‐α‐helical globins (e.g., hemoglobin (Hb) and myoglobin (Mb)) play pivotal roles in ligand (e.g., O_2_) transport, storage, and sensing, as well as in heme‐Fe‐based catalysis 1, 2, 3, 4, 5, 6, 7. Most of them display the classical 3/3 globin fold, which is made up by six α‐helices facing the heme; the A, B, and E α‐helices form one face of the sandwich, the other side being built by the F, G, and H α‐helices 1, 3, 4, 7, 8, 9. Recently, the 2/2 subset of the classical 3/3 α‐helical fold was discovered; it is a sort of bundle composed of antiparallel pairs, the α‐helices B/E and G/H sandwiching the heme 10, 11. In all‐α‐helical globins, the heme is deeply buried in the protein matrix contacting several hydrophobic residues that prevent the oxidation of the metal center [1, 3, 4, 5, 7, 11]. The fifth coordination ligand of the heme‐Fe atom is invariantly the side chain of the proximal HisF8 residue 1, 3, 4, 7. The heme distal ligand is represented generally by the E7 residue (mostly His and Tyr), which contributes to the modulation of the metal center reactivity and the stability of the heme‐bound ligand 1, 3, 4, 7, 12, 13, 14, 15, 16, 17.

Over the last two decades, monomeric all‐β‐barrel heme‐proteins have been reported. They include *Rhodnius prolixus* nitrophorins (NPs) 18, 19, 20, 21, and nitrobindins (Nbs), spanning from bacteria to *H. sapiens* 21, 22, 23, 24. Furthermore, the mixed α‐helical‐β‐barrel heme‐proteins human α1‐microglobulin and *Cimex lectularius* NP have been described 21, 25, 26, 27. Nbs display a ten‐stranded antiparallel β‐barrel fold in which the penta‐coordinated heme‐Fe atom is secured to the protein by the proximal His residue 21, 22, 23, 24, 27. In Nbs, the heme is highly solvent exposed and is stable in the ferric form, allowing to bind NO 21, 22, 23, 24. Interestingly, human Nb has been described as a domain of the nuclear protein named THAP4 whose function is still unknown 21, 23, 24. THAP4 is composed of 567 amino acids and consists of an N‐terminal modified zinc finger domain that binds DNA and the *C*‐terminal Nb(III) domain 23, 28, 29.

Here, the first evidence of the heme‐Fe‐based detoxification of peroxynitrite by the ferric all‐β‐barrel *C*‐terminal Nb(III) domain of THAP4 (hereafter human Nb(III)) is reported. Human Nb(III) catalyzes efficiently the conversion of peroxynitrite to ${\text{NO}}_{3}^{-}$ and impairs the peroxynitrite‐mediated nitration of free l‐tyrosine. These results point to a role of THAP4 in reactive nitrogen species chemistry.

## Materials

The pReceiver‐B03 vector containing the transcript variant 2 of *H. sapiens* Nb(III) domain (GeneCopoeia, Rockville, MD, USA) was used to amplify by PCR the Nb gene (fw_HindIII_NdeI: 5′‐GCCCAAGCTTCATATGGAGCCCCCCAAG‐3′ and rv_BamHI: 5′‐CGCGGATCCTTACGGGGTCAC‐3′). The fragment of 500 bp was first subcloned in the pBluescript KS(−) and finally cloned in the pET‐28a (+) vector. The *Escherichia coli* BL21(DE3) strain was used to express the 6 × His‐tag‐Nb in the presence of 0.2 mm δ‐aminolevulinic acid. The expression of the 6 × His‐tagged Nb was induced by adding 1 mm isopropyl‐β‐d‐thiogalactoside for 16 h at 37 °C. The bacterial pellet was lysed in 20 mm phosphate buffer pH 7.5, 140 mm NaCl, and 0.015% Tween‐20, and the supernatant was loaded onto a His‐Trap affinity chromatography column (GE Healthcare Bio‐Sciences, Amersham, UK). The adsorbed 6 × His‐tag‐Nb was eluted by a linear gradient of imidazole (20 mm phosphate buffer pH 7.4, 500 mm NaCl, and 10–1000 mm imidazole). The fractions containing the fusion protein were dialyzed against 20 mm phosphate buffer, pH 7.4, and analyzed by western blot using the primary anti‐6 × His‐tag antibody (Thermo Fisher Scientific, Waltham, MA, USA). The human Nb(III) concentration was determined spectrophotometrically by the pyridine hemochromogen method 30. Human apo‐Nb was prepared by the acid–acetone method 30.

Peroxynitrite was purchased from Cayman Chemical (Ann Arbor, MI, USA). The concentration of peroxynitrite was determined spectrophotometrically prior to each experiment by measuring the absorbance at 302 nm (ε = 1.705 × 10^3^ m ^−1^·cm^−1^) 31. l‐Tyrosine, nitro‐l‐tyrosine, and 1,1‐diethyl‐2‐hydroxy‐2‐nitroso‐hydrazine (NONOate) were obtained from Sigma‐Aldrich (St. Louis, MO, USA). All the other chemicals were purchased from Merck KGaA (Darmstadt, Germany). All chemicals were of analytical grade and were used without further purification.

## Methods

Peroxynitrite isomerization by human Nb(III) and apo‐Nb was investigated by rapid mixing the human Nb(III) or apo‐Nb solutions (final concentration ranging between 5.0 × 10^−6^ and 3.5 × 10^−5^ m) with the peroxynitrite solution (final concentration, 2.0 × 10^−4^ m). Kinetics were recorded by using the SFM‐20/MOS‐200 rapid‐mixing stopped‐flow apparatus (BioLogic Science Instruments, Claix, France) monitoring absorbance changes at 302 nm 31; the light path of the observation chamber was 10 mm, and the dead‐time was 1.3 ms. In agreement with literature data 31, 32, the absorbance at 302 nm decreased upon mixing the human Nb(III) and peroxynitrite solutions, reflecting the isomerization of peroxynitrite. No absorbance spectroscopic changes were observed in the Soret region in the course of the human Nb(III)‐mediated isomerization of peroxynitrite.

**Figure 1:**
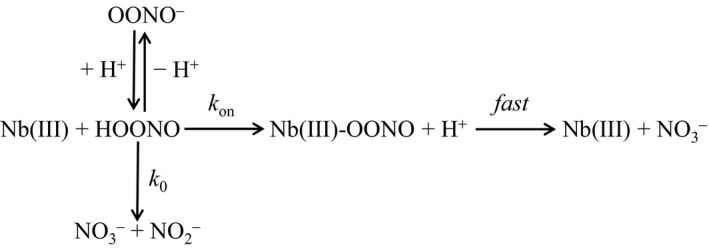
Peroxynitrite isomerization in the absence and presence of human Nb(III).

Kinetics of peroxynitrite isomerization by human Nb(III) and apo‐Nb were analyzed in the framework of the reaction scheme shown in Fig. 1 31, 32, 33, 34, 35, 36, 37, 38, 39, 40, 41, 42, 43, 44, 45, 46.

Values of the pseudo‐first‐order rate constant for peroxynitrite isomerization in the presence of human Nb(III) and human apo‐Nb (i.e., *k*_obs_) were determined from the analysis of the time‐dependent absorbance decrease at 302 nm, according to Eqn (1) 36, 37, 38, 39, 40, 41, 42, 43, 44, 45, 46:

(1) $${\left[\text{peroxynitrite}\right]}_{t}={\left[\text{peroxynitrite}\right]}_{i}\times e^{-k_{\mathrm{obs}}\times t}$$

Values of the second‐order rate constant for peroxynitrite isomerization by human Nb(III) (i.e., *k*_on_) and of the first‐order rate constant for the spontaneous decay of peroxynitrite (i.e., *k*_0_) were obtained from the dependence of *k*_obs_ on the ferric heme‐protein concentration (i.e., [human Nb(III)], according to Eqn (2) 36, 37, 38, 39, 40, 41, 42, 43, 44, 45, 46:

(2) $$k_{\mathrm{obs}}=k_{\mathrm{on}}\times \left[\text{human}\;\;\text{Hb}\left(\text{III}\right)\right]+k_{0}$$

The effect of pH on values of *k*_on_ and *k*_0_ for peroxynitrite isomerization was analyzed according to Eqn (3) 36, 38, 39, 46, 47, 48:

(3) $$k=\left(k_{\lim}\times 10^{-\mathrm{pH}}\right)/\left(10^{-\mathrm{pH}}+10^{-pKa}\right)$$

where *k* is either *k*_obs_ or *k*_0_ and *k*_lim_ represents the top asymptotic value of *k* under conditions where pH ≪ p*K*_a_.

The reaction of peroxynitrite with free l‐tyrosine was carried out at pH 7.1 and 25.0 °C by adding 0.2 mL of an alkaline (1.0 × 10^−3^ m NaOH), ice‐cooled solution of peroxynitrite (2.0 × 10^−3^ m) to 1.8 mL of a buffered (5.0 × 10^−2^ m phosphate buffer) solution of l‐tyrosine (final concentration, 1.0 × 10^−4^ m) in the absence and presence of human Nb(III) and apo‐Nb (final concentration, 3.5 × 10^−5^ m). The amount of nitro‐l‐tyrosine was determined by HPLC analysis 36, 37, 38, 39, 46.

The ${\text{NO}}_{2}^{-}$ and ${\text{NO}}_{3}^{-}$ concentrations were determined spectrophotometrically at 543 nm by using the Griess reagent and VCl_3_ to catalyze the conversion of ${\text{NO}}_{3}^{-}$ to ${\text{NO}}_{2}^{-}$ 36, 39, 49. The samples were prepared by mixing 0.5 mL of either a human Nb(III) or an apo‐Nb solution (final concentration, 3.5 × 10^−5^ m in 5.0 × 10^−2^ m phosphate buffer, pH 7.1) with 0.5 mL of a peroxynitrite solution (final concentration, 2.0 × 10^−4^ m in 1.0 × 10^−2^ m NaOH) while vortexing, at 25.0 °C. The reaction mixture was analyzed within 10 min according to literature 36, 39, 49.

The absorbance spectrum of nitrosylated human Nb(III) was obtained by adding NONOate (1.0 × 10^−4^) m to human Nb(III) (3.5 × 10^−6^ m).

Kinetic data were analyzed using the MATLAB program (The MathWorks Inc., Natick, MA, USA). The results are given as mean values of at least four experiments plus and minus the standard deviation.

## Results and Discussion

**Figure 2:**
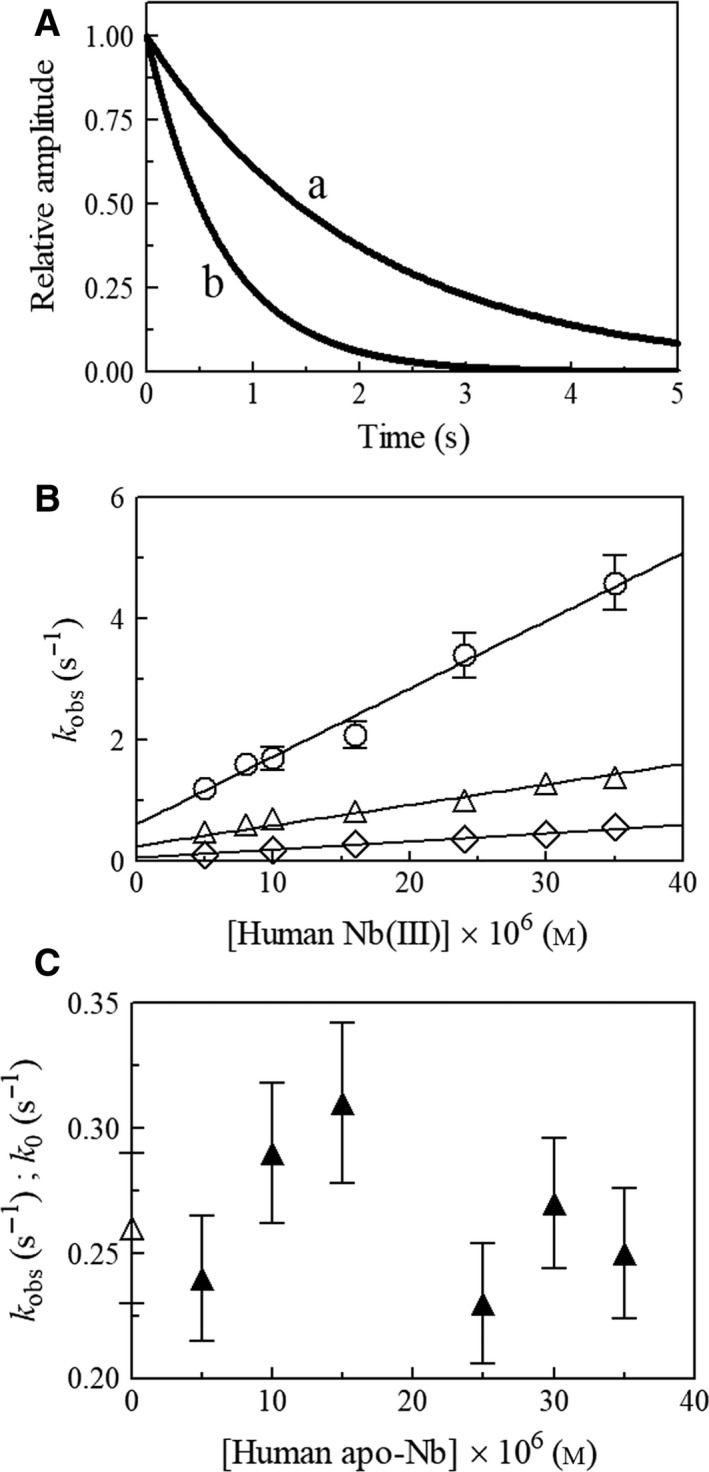
Effect of human Nb(III) on peroxynitrite isomerization (*k*_obs_), at 25.0 °C. (A) Averaged time courses of the human Nb(III)‐mediated isomerization of peroxynitrite at pH 7.1. Data analysis according to Eqn (1) allowed us to determine the following values of *k*_obs_: 4.9 × 10^−1^ s^−1^ (trace a) and 1.4 s^−1^ (trace b). The human Nb(III) concentration was 5.0 × 10^−6^ m (trace a) and 3.5 × 10^−5^ m (trace b). The peroxynitrite concentration was 2.0 × 10^−4^ m. (B) Dependence of *k*_obs_ on the human Nb(III) concentration, at pH 6.1 (open circles), 7.1 (open triangles), and 7.7 (open diamonds). Kinetics were analyzed according to Eqn (2) with values of *k*_obs_ and *k*_0_ given in Table 1. (C) Dependence of *k*_obs_ on the human apo‐Nb concentration, at pH 7.1. The symbol on the ordinate axis indicates the value of *k*_0_ (=(2.6 ± 0.3) × 10^−1^ s^−1^). The average value of *k*_obs_ is (2.7 ± 0.4) × 10^−1^ s^−1^. The peroxynitrite concentration was 2.0 × 10^−4^ m. Where not shown, the standard deviation is smaller than the symbol.

Under all the experimental conditions, most of the time course of peroxynitrite isomerization (from 92% to 100%) was fitted to a single‐exponential decay according to Eqn ((1); Fig. 2, panel A). In fact, < 10% of the initial part of the time course of peroxynitrite isomerization was lost in the dead‐time of the rapid‐mixing stopped‐flow apparatus, depending on the decomposition rate.

The pseudo‐first‐order rate constant for human Nb(III)‐mediated isomerization of peroxynitrite (i.e., *k*_obs_) increases linearly with the protein concentration (Fig. 2, panel B). This suggests that (a) the formation of the transient human Nb(III)‐OONO species represents the rate‐limiting step in catalysis, and (b) the conversion of human Nb(III)‐OONO to Nb(III) and ${\text{NO}}_{3}^{-}$ and ${\text{NO}}_{2}^{-}$ is faster than human Nb(III)‐OONO formation by at least 10‐fold. The analysis of the data shown in Fig. 2 (panel B), according to Eqn (2), allowed us to determine the values of the second‐order rate constant for peroxynitrite isomerization by human Nb(III) (i.e., *k*_on_, corresponding to the slope of the linear plots) and of the first‐order rate constant for the spontaneous peroxynitrite isomerization (i.e., *k*_0_, corresponding to the *y*‐intercept of the linear plots) (Table 1). Values of *k*_0_ here determined agree with those previously reported [32, 36, 37, 38, 39, 40, 41, 42, 45, 46].

**Table 1:** Values of *k*_on_ and *k*_0_ values for peroxynitrite isomerization by human Nb(III), at 25.0 °C

pH	*k*_on_ (m ^−1^·s^−1^)	*k*_0_ (s^−1^)
6.1	(1.1 ± 0.1) × 10^5^	(6.2 ± 0.7) × 10^−1^
6.3	(9.8 ± 1.1) × 10^4^	(4.9 ± 0.5) × 10^−1^
6.6	(8.1 ± 0.8) × 10^4^	(4.1 ± 0.4) × 10^−1^
7.1	(3.4 ± 0.4) × 10^4^	(2.6 ± 0.3) × 10^−1^
7.4	(1.8 ± 0.2) × 10^4^	(1.3 ± 0.1) × 10^−1^
7.7	(1.3 ± 0.2) × 10^4^	(6.3 ± 0.8) × 10^−2^

To confirm the role of the heme‐Fe(III) atom in catalysis, values of *k*_obs_ have been determined in the presence of human apo‐Nb, which does not catalyze the peroxynitrite isomerization. Indeed, values of *k*_obs_ obtained in the presence of human apo‐Nb correspond to those of *k*_0_ (Fig. 2, panel C) as reported, among others, for horse heart apo‐Mb and human apo‐Hb 36.

In the presence of human Nb(III), the values of the relative yield of ${\text{NO}}_{3}^{-}$ and ${\text{NO}}_{2}^{-}$ for the isomerization of peroxynitrite are 89 ± 2% and 12 ± 1%. However, in the absence of human Nb(III) and in the presence of human apo‐Nb, the values of the relative yield of ${\text{NO}}_{3}^{-}$ and ${\text{NO}}_{2}^{-}$ are 68 ± 3% and 31 ± 2%, and 71 ± 2% and 28 ± 3%, respectively. These data well agree with those reported for peroxynitrite isomerization by ferric heme‐proteins such as horse heart apo‐Mb and human apo‐Hb 36.

**Figure 3:**
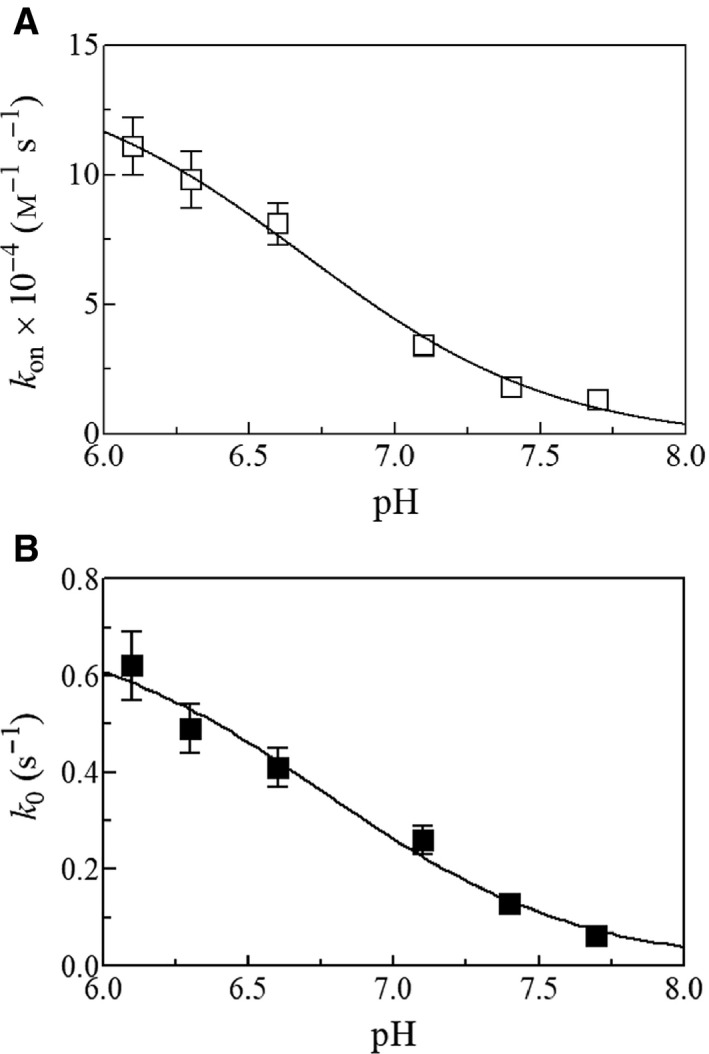
Effect of pH on human Nb(III)‐mediated peroxynitrite isomerization, at 25.0 °C. (A) Effect of pH on values of *k*_on_. The continuous line was calculated according to Eqn (3) with p*K*_a_ = 6.7 ± 0.2 and *k*_lim_ = (1.4 ± 0.1) × 10^5^ m ^−1^·s^−1^. (B) Effect of pH on values of *k*_0_. The continuous line was calculated according to Eqn (3) with p*K*_a_ = 6.8 ± 0.2 and *k*_lim_ = (7.1 ± 0.5) × 10^−1^ s^−1^. Where not shown, the standard deviation is smaller than the symbol.

The pH dependence of *k*_on_ and *k*_0_ values for peroxynitrite isomerization allowed us to identify tentatively the species that preferentially react(s) with the heme‐Fe(III) atom. Values of *k*_on_ and *k*_0_ increase upon decreasing pH (Fig. 3, panels A and B). The p*K*_a_ values for the pH dependence of *k*_on_ and of *k*_0_ are 6.7 ± 0.2 and 6.8 ± 0.2, respectively (Fig. 3). The p*K*_a_ values here determined agree well with those previously reported for the heme‐protein‐mediated isomerization of peroxynitrite 31, 33, 39, 45, 46.

The close similarity of the pH dependence of *k*_on_ for the human Nb(III)‐mediated isomerization of peroxynitrite (Fig. 3, panel A) and of *k*_0_ for peroxynitrite isomerization in the absence of human Nb(III) (Fig. 3, panel B) suggests that the HOONO species (Fig. 1) reacts preferentially with the heme‐Fe(III) atom 31, 33, 38, 46. Since the absorbance spectrum of Nb(III) is unaffected by pH over the whole range explored (i.e., between pH 6.1 and 7.7; Fig. S1), it is unlikely that values of *k*_on_ (Fig. 3, panel A) are affected by the acid–base equilibrium(a) of the ferric heme‐protein.

**Figure 4:**
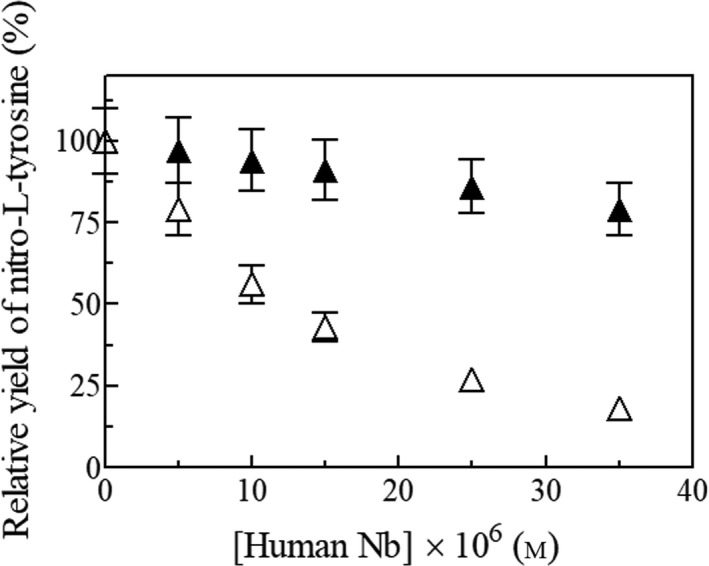
Protective role of Nb on peroxynitrite‐mediated nitrosylation of free L‐tyrosine, at pH 7.1 and 25.0 °C. Effect of human Nb(III) (open triangles) and apo‐Nb (filled triangles) concentration on the relative yield of nitro‐l‐tyrosine formed by the reaction of peroxynitrite with free l‐tyrosine. The symbol on the ordinate axis (open triangle) indicates the relative yield of nitro‐l‐tyrosine obtained in the absence of human Nb(III) and apo‐Nb. The free l‐tyrosine concentration was 1.0 × 10^−4^ m. The peroxynitrite concentration was 2.0 × 10^−4^ m. Relative nitro‐l‐tyrosine yield (%) = (yield with added human Nb(III) or apo‐Nb/yield with no human Nb(III) or apo‐Nb) × 100. Where not shown, the standard deviation is smaller than the symbol.

To analyze the protective role of human Nb(III) against peroxynitrite‐mediated nitration, the relative yield of nitro‐l‐tyrosine formed by the reaction of peroxynitrite with free l‐tyrosine in the absence and presence of human Nb(III) and apo‐Nb was determined. As expected, human Nb(III) protects dose‐dependently free l‐tyrosine against peroxynitrite‐mediated nitration, whereas l‐tyrosine nitration is not prevented by human apo‐Nb (Fig. 4).

**Figure 5:**
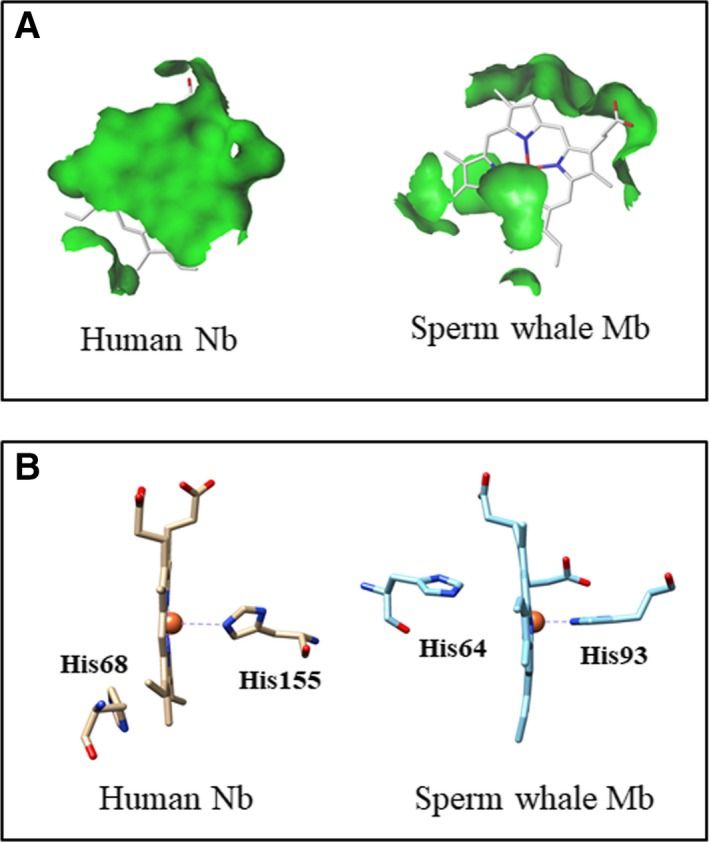
Structural aspects of the heme site of human Nb(III) (PDB code: 3IA8) 23 and sperm whale Mb(III) (PDB code: 5MBN) 50. (A) Solvent accessible surface of the heme distal site of human Nb(III) and sperm whale Mb(III). The heme distal plane is much more solvent accessible in human Nb(III) than in sperm whale Mb(III). (B) Schematic representation of the heme coordination in human Nb(III) and sperm whale Mb(III). The heme proximal residues of human Nb and sperm whale Mb are His155 and His93, respectively. In human Nb(III) and sperm whale Mb(III), the heme‐Fe(III) atom is positioned ~ 0.35 to ~ 0.65 Å out‐of‐plane on the proximal side with respect to the pyrrole nitrogen atoms of the porphyrin, respectively. The pictures have been drawn with UCSF‐Chimera package 64.

The value of *k*_on_ for peroxynitrite isomerization by all‐β‐barrel human Nb(III) is similar to that of most ferric all‐α‐helical globins (i.e., *Methanosarcina acetivorans* protoglobin (*Ma*‐Pgb), *Mycobacterium tuberculosis* truncated hemoglobin N (*Mt*‐trHbN)*, Pseudoalteromonas haloplanktis* truncated hemoglobin O (*Ph*‐trHbO), horse heart Mb(III), sperm whale Mb(III), human Hb(III), human serum heme‐albumin (human SA‐heme), and *Fusarium oxysporum* cytochrome P450 NO reductase) and mixed β‐barrel/α‐helical cardiolipin‐bound and carboxymethylated cytochrome *c* (CL‐cyt*c* and CM‐cyt*c*, respectively) (Table 2) (33, 36, 37, 38, 39, 40, 41, 42, 43, 44 and present study), suggesting that neither the very different structural organization 1, 3, 22, 23, 50, 51, 52, 53, 54, 55, 56, 57, 58 nor the different solvent and ligand accessibility to the metal center (Fig. 5, panel A) (1, 3, 22, 23, 50, 51, 52, 53, 54, 55, 56, 57, 58 and present study) nor the Lewis acidity of the heme‐Fe(III) atom 38 are at the root of the modulation of peroxynitrite isomerization. In fact, the reactivity of these heme‐proteins appears to be limited by the out‐to‐in‐plane movement of the heme‐Fe(III) atom preceding ligand (i.e., peroxynitrite) binding (Fig. 5, panel B). Of note, in ferric all‐β‐barrel human Nb and in most all‐α‐helical globins (e.g., sperm whale Mb and human Hb), the heme‐Fe(III) atom is positioned ~ 0.35 and ~ 0.65 Å out‐of‐plane on the proximal side with respect to the pyrrole nitrogen atoms of the porphyrin, respectively (Fig. 5, panel B) ([1, 3, 22, 23, 50, 51, 52, 53, 54, 57, 58] and present study). The high reactivity of ferric *Campylobacter jejuni* truncated hemoglobin P (*Cj*‐trHbP) (Table 2) reflects the high ligand accessibility to the heme center by the HisE7 path, the dynamic balance of hydrogen‐bonding interactions at the heme distal site, and the penta‐coordination of the heme‐Fe atom; this suggested a role of *Cj*‐trHbP in performing a peroxidase‐like chemistry 46, 59, 60, 61. Furthermore, peroxynitrite isomerization by penta‐coordinated sterically open heme‐model compounds (Table 2) could reflect the in‐ or out‐of‐plane position of the heme‐Fe(III) atom on the proximal side with respect to the pyrrole nitrogen atoms of the macrocycle 34, 35, 45.

**Table 2:** Peroxynitrite scavenging by ferric heme‐proteins and heme‐model compounds. n.d., not determined

Heme‐protein	*k*_on_ (m ^−1^·s^−1^)	*k*_0_ (s^−1^)
*Methanosarcina acetivorans* Pgb^a^	3.8 × 10^4^	2.8 × 10^−1^
*Mycobacterium tuberculosis* trHbN^b^	6.2 × 10^4^	2.7 × 10^−1^
*Pseudoalteromonas haloplanktis*‐trHbO^c^	2.9 × 10^4^	2.8 × 10^−1^
*Campylobacter jejuni*‐trHbP d	9.6 × 10^5^	3.0 × 10^−1^
Horse heart Mb^e^	2.9 × 10^4^	3.5 × 10^−1^
Sperm whale Mb^f^	1.6 × 10^4^	n.d.
Human Hb^e^	1.2 × 10^4^	3.0 × 10^−1^
Human Nb^g^	3.4 × 10^4^	2.6 × 10^−1^
Human SA‐heme^h^	4.1 × 10^5^	2.6 × 10^−1^
CL‐cyt*c* i	3.2 × 10^5^	2.9 × 10^−1^
CM‐cyt*c* j	6.8 × 10^4^	2.8 × 10^−1^
*Fusarium oxysporum* cytochrome P450 NO reductase^k^	~ 5 × 10^5^	9.0 × 10^−2^
Fe(TMPS)^l^	6.0 × 10^4^	5.5 × 10^−1^
Fe(TMPS)^m^	3.0 × 10^5^	1.35
Fe(TPPS)^m^	8.6 × 10^5^	1.35
Fe(TMPyP)^m^	1.6 × 10^6^	1.35
MP11^n^	4.1 × 10^4^	2.8 × 10^−1^

a pH 7.4 and 20.0 °C. From 44.

b pH 7.0 and 20.0 °C. From 42.

c pH 7.0 and 20.0 °C. From 43.

d pH 7.3 and 25.0 °C. From 46.

e pH 7.0 and 20.0 °C. From 36.

f pH 7.5 and 20.0 °C. From 38.

g pH 7.1 and 25.0 °C. Present study.

h pH 7.2 and 22.0 °C. From 39.

i pH 7.0 and 20.0 °C. CL was 1.6 × 10^−4^ m. From 40.

j pH 7.0 and 20.0 °C. From 41.

k pH 8.0 and 12.0 °C. From 33.

l pH 7.6 and 25.0 °C. From 34.

m pH 7.4 and 37.0 °C. From 35.

n pH 7.2 and 20.0 °C. From 45.

Human Nb(III) not only catalyzes the detoxification of peroxynitrite but also binds reversibly NO (Fig. S1), as already reported for *Arabidopsis thaliana* Nb 22. In fact, upon mixing human Nb(III) and NONOate solutions, the maximum of the absorbance spectrum of human Nb(III) shifts from 406 nm (human Nb(III)) to 412 nm (human Nb(III)‐NO). Moreover, human Nb(III)‐NO can be converted to human Nb(III) by pumping off NO.

In light of these considerations, data here reported highlight for the first time the capability of the Nb‐like domain of human THAP4 protein to catalyze peroxynitrite scavenging, to impair the peroxynitrite‐mediated nitration of free l‐tyrosine, and to bind NO. Considering the structural organization of THAP4 23, 28, 29, it can be speculated that THAP4 may play a role in the chemistry of reactive nitrogen species by coupling the heme‐based Nb reactivity with the modulation of genes transcription. This somehow resembles other heme‐proteins like NPAS2, in which the heme redox status controls NPAS heterodimerization with BMAL1 and, in turn, DNA binding and target gene expression 62, 63. Similar to *A. thaliana* Nb 22, human Nb is firmly in the ferric form possibly distinguishing among NO, CO, and O_2_. In fact, human Nb(III) selectively binds NO without recognizing CO and O_2_ that are typical diatomic gaseous ligands of ferrous metal centers.

We are presently working on the functional analysis of the full‐length human THAP4 protein as well as of its N‐ and/or C‐terminal deleted forms to understand the molecular mechanisms underpinning THAP4 cellular functions.

## Author contributions

GDS performed the experiments and analyzed data. AM performed the comparative analysis of data. FP contributed to analyze the results and to draft the paper. PA coordinated the study and wrote the paper.

## Conflict of interest

The authors declare no conflict of interest.

## Supporting information


**Fig. S1.** Absorbance spectra of human Nb(III) and Nb(III)‐NO (*T* = 25.0 °C). (A) Absorbance spectra of human Nb(III) at pH 6.1 (spectrum a) and 7.8 (spectrum b). For clarity, the absorbance spectrum obtained at pH 7.8 has been up‐shifted of 0.5 units. (B) Absorbance spectra of human Nb(III) (continuous line) and Nb(III)‐NO (dashed line) at pH 7.4. The λ_max_ values of human Nb(III) and Nb(III)‐NO are 406 and 412 nm, respectively.
