# Supplementary material for: Human nitrobindin: the first example of an all‐β‐barrel ferric heme‐protein that catalyzes peroxynitrite detoxification
Source: FEBS Open Bio. 2018 Nov 9;8(12):2002–10. doi: 10.1002/2211-5463.12534 (PMC6275384; doi:10.1002/2211-5463.12534)
Supplement: Supplementary file 1 — Fig. S1. Absorbance spectra of human Nb(III) and Nb(III)‐NO (T = 25.0 °C). (A) Absorbance spectra of human Nb(III) at pH 6.1 (spectrum a) and 7.8 (spectrum b). For clarity, the absorbance spectrum obtained at pH 7.8 has been up‐shifted of 0.5 units. (B) Absorbance spectra of human Nb(III) (continuous line) and Nb(III)‐NO (dashed line) at pH 7.4. The λmax values of human Nb(III) and Nb(III)‐NO are 406 and 412 nm, respectively. [file FEB4-8-2002-s001.DOCX]

**Supplementary Materials**

**Human nitrobindin: the first example of an all-β-barrel ferric heme-protein that catalyzes peroxynitrite detoxification**

**Giovanna De Simone, ^1^ Alessandra di Masi, ^1^ Fabio Polticelli, ^1,2^ and Paolo Ascenzi ^3,^***

^1^ Department of Sciences, Roma Tre University, I-00146, Roma, Italy

^2^ National Institute of Nuclear Physics, Roma Tre Section, I-00146, Roma, Italy

^3^ Interdepartmental Laboratory for Electron Microscopy, Roma Tre University,

I-00146 Roma, Italy

**Running title**: Peroxynitrite scavenging by human nitrobindin domain of THAP4 protein

**Keywords**: Human nitrobindin; peroxynitrite scavenging; protection of L-tyrosine nitration; kinetics.

**Abbreviations**: human Nb(III), ferric human nitrobindin; Nb(III)-NO, nitrosylated human Nb(III).

* **Corresponding author**: Paolo Ascenzi, Interdepartmental Laboratory for Electron Microscopy, Roma Tre University, Via della Vasca Navale 79, I-00146 Roma, Italy.

Tel: +39-06-57333621; Fax: +39-06-57336321; E-mail address: [ascenzi@uniroma3.it](mailto:ascenzi@uniroma3.it)

**Fig. S1**. Absorbance spectra of human Nb(III) and Nb(III)-NO (*T* = 25.0 °C). (A) Absorbance spectra of human Nb(III) at pH 6.1 (spectrum a) and 7.8 (spectrum b). For clarity, the absorbance spectrum obtained at pH 7.8 has been up-shifted of 0.5 units. (B) Absorbance spectra of human Nb(III) (continuous line) and Nb(III)-NO (dashed line) at pH 7.4. The λ_max_ values of human Nb(III) and Nb(III)-NO are 406 nm and 412 nm, respectively.
